# NXN Gene Epigenetic Changes in an Adult Neurogenesis Model of Alzheimer’s Disease

**DOI:** 10.3390/cells11071069

**Published:** 2022-03-22

**Authors:** Idoia Blanco-Luquin, Blanca Acha, Amaya Urdánoz-Casado, Eva Gómez-Orte, Miren Roldan, Diego R. Pérez-Rodríguez, Juan Cabello, Maite Mendioroz

**Affiliations:** 1 Neuroepigenetics Laboratory-Navarrabiomed, Hospital Universitario de Navarra (HUN), Universidad Pública de Navarra (UPNA), IdiSNA (Navarra Institute for Health Research), 31008 Pamplona, Spain; blanca.acha.santamaria@navarra.es (B.A.); amaya.urdanoz.casado@navarra.es (A.U.-C.); mroldana@navarra.es (M.R.); maitemendilab@gmail.com (M.M.); 2 CIBIR (Center for Biomedical Research of La Rioja), 26006 Logroño, Spain; emgomez@riojasalud.es (E.G.-O.); juan.cabello@riojasalud.es (J.C.); 3 Neurophysiology Department, Hospital Universitario de Navarra (HUN), IdiSNA (Navarra Institute for Health Research), 31008 Pamplona, Spain; drpr25@gmail.com; 4 Department of Neurology, Hospital Universitario de Navarra (HUN), IdiSNA (Navarra Institute for Health Research), 31008 Pamplona, Spain

**Keywords:** adult hippocampal neurogenesis, NPCs, Alzheimer’s disease, Aβ peptide, DNA methylation, gene expression, *NXN*, *CNTNAP1*, *SEPT5-GP1BB*, *TBX5*

## Abstract

In view of the proven link between adult hippocampal neurogenesis (AHN) and learning and memory impairment, we generated a straightforward adult neurogenesis *in vitro* model to recapitulate DNA methylation marks in the context of Alzheimer’s disease (AD). Neural progenitor cells (NPCs) were differentiated for 29 days and Aβ peptide 1–42 was added. mRNA expression of Neuronal Differentiation 1 (*NEUROD1*), Neural Cell Adhesion Molecule 1 (*NCAM1*), Tubulin Beta 3 Class III (*TUBB3*), RNA Binding Fox-1 Homolog 3 (*RBFOX3*), Calbindin 1 (*CALB1*), and Glial Fibrillary Acidic Protein (*GFAP*) was determined by RT-qPCR to characterize the culture and framed within the multistep process of AHN. Hippocampal DNA methylation marks previously identified in Contactin-Associated Protein 1 (*CNTNAP1*), SEPT5-GP1BB Readthrough (*SEPT5-GP1BB*), T-Box Transcription Factor 5 (*TBX5*), and Nucleoredoxin (*NXN*) genes were profiled by bisulfite pyrosequencing or bisulfite cloning sequencing; mRNA expression was also measured. *NXN* outlined a peak of DNA methylation overlapping type 3 neuroblasts. Aβ-treated NPCs showed transient decreases of mRNA expression for *SEPT5-GP1BB* and *NXN* on day 9 or 19 and an increase in DNA methylation on day 29 for *NXN*. *NXN* and *SEPT5-GP1BB* may reflect alterations detected in the brain of AD human patients, broadening our understanding of this disease.

## 1. Introduction

Adult neurogenesis (AN) is the process of forming functional neurons *de novo*. In the adult mammalian brain, neurogenesis occurs predominantly in specific brain niches: the subgranular zone (SGZ) of the dentate gyrus (DG) of the hippocampus and the subventricular zone (SVZ) lining the lateral ventricles [1,2]. During the process of adult hippocampal neurogenesis (AHN), neural stem cells (NSCs) self-renew and differentiate, giving rise to transient amplifying progenitors (TAPs), neuroblasts, and eventually mature neurons, astrocytes, and oligodendrocytes.

AHN regulators can be divided into intrinsic or extrinsic factors, that is, transcription factors (TFs) synthesized by the developing neural precursors and neurons, and growth factors and neurotrophins secreted from the surrounding niche, respectively [3]. Epigenetic mechanisms tightly regulate extrinsic and intrinsic factors [4], controlling both temporal and spatial gene expression. Sequential steps of AN are regulated directly or indirectly by *de novo* methylation and maintenance of methylation marks [5]. Each distinct human brain region (cerebral cortex, cerebellum, and pons) has a characteristic DNA methylation signature [6], and even within brain regions such as the hippocampus, global methylation varies between neuronal subtypes [7].

During both physiological and pathological aging in humans, AHN clearly emerges as a robust phenomenon [8]. AHN is involved with the normal functionality of hippocampal circuits, which demonstrates an important link between AN and cognitive processes [9]. Thus, impaired neurogenesis may negatively impact the survival of adult-born neurons and contribute to learning and memory failure, as occurs with aging and neurological disorders, e.g., Alzheimer’s disease (AD) [8,10,11].

AD is the most common neurodegenerative disorder, characterized by progressive memory loss and cognitive decline caused by widespread loss of neurons and synaptic connections in the cortex, hippocampus, amygdala, and basal forebrain, and by a gradually significant loss of brain mass. The amyloid precursor protein (APP) plays a key role in normal brain development by influencing NSC proliferation, cell fate specification, and neuronal maturation [10]. However, its derivative, the amyloid β (Aβ) peptide, a cleavage product of the APP enzymatic processing, is the major component of amyloid plaques, one of the hallmark pathologies found in brains of AD patients. Monomeric Aβ can self-aggregate to form oligomers, protofibrils, and amyloid fibrils, which deposit as amyloid plaques. Although the impact of Aβ on neurogenesis is still controversial, it is well known that Aβ plaques can cause severe damage to neurons and astrocytes, which results in the gradual loss of neurons associated with AD symptoms [11].

Remarkable alterations in AHN have been detected at early stages of AD, even before the onset of hallmark lesions or neuronal loss [8,12]. Impairments in epigenetic mechanisms lead to the generation of damaged neurons from NSCs, exacerbating the loss of neurons and deficits in learning and memory that characterize AD pathology [11]. Indeed, we and others have described epigenetic changes in DNA methylation in the hippocampus of AD patients at the genome-wide level [6,13]. In a previous study, we reported altered DNA methylation in the AD hippocampus occurring at specific regulatory regions crucial for neuronal differentiation; moreover, a set of neurogenesis-related genes were identified in the damaged tissue [6]. Hence, a better understanding of AHN impairment observed at the initial and later stages of AD by noninvasive methods may reveal insights into the pathogenesis of AD. What is more, restoration of normal levels of AHN may provide a potential therapeutic strategy to delay or halt AD-linked cognitive decline [8,12].

Here, we propose an intuitive *in vitro* approach to assess a stepwise lineage progression, as occurs during *in vivo* neurogenesis, by using human neural progenitor cells (NPCs) derived from an induced pluripotent stem cell (iPSC) line as the starting source material. In order to infer whether the differentiation of human NPCs into mature neurons is disrupted in the AD microenvironment, we designed an observational descriptive study by generating an *in vitro* model triggered by prolonged exposure to nanomolar concentrations of Aβ peptide 1–42. Next, we evaluated DNA methylation levels and mRNA expression changes of specific neurogenesis-related candidate genes.

## 2. Materials and Methods

### 2.1. NPCs Culture, Neuronal Differentiation and Aβ Peptide Administration

NPCs Derived from XCL1 DCXpGFP (ACS5005™, American Type Culture Collection, ATCC, Manassas, VA, USA) were cultured following manufacturer recommendations. Briefly, 0.30 × 10^6^ NPCs were seeded onto a CellMatrix Basement Membrane Gel (ATCC^®^ ACS3035™) coated 12-well plate and incubated in NPC expansion medium: complete growth medium including DMEM/F-12 (Gibco, Fisher Scientific, Waltham, MA, USA), supplemented with the Growth Kit for Neural Progenitor Cell Expansion (ATCC^®^ ACS3003) and then maintained in a humidified incubator (5% CO_2_, 37 °C).

Neuronal differentiation experiments were carried out for 9, 19, and 29 days by plating NPCs at a seeding density of 80,000 viable cells/cm^2^ in 6-well coated culture plates. First, NPCs were incubated in an expansion medium (day 0). From day 1 (post-seeding), half of the medium was changed for differentiation medium every 2–3 days throughout the duration of the culture period. Complete Differentiation Medium consisted of serum-free neuronal basal BrainPhys™ Neuronal Medium, formulated to improve the electrophysiological and synaptic properties of the neurons [14], NeuroCult™ SM1 Neuronal Supplement (1:50), N2 Supplement-A (1:100), Recombinant Human Brain-Derived Neurotrophic Factor (BDNF, 20 ng/mL), Recombinant Human Glial-Derived Neurotrophic Factor (GDNF, 20 ng/mL), Dibutyryl-cAMP (1 mM) and ascorbic acid (200 nM) (STEMCELL Technologies, Vancouver, BC, Canada). Half-fresh medium containing Aβ protein fragment 1–42 (50 nM; Sigma-Aldrich, St. Louis, MO, USA) or DMSO (Sigma-Aldrich) as a vehicle was added once a week.

NPCs were harvested on day 0 and 9, 19, and 29 days of differentiation for both conditions by detaching them with Accutase (Innovative Cell Technologies, San Diego, CA, USA), then washed with Dulbecco’s phosphate-buffered saline (DPBS, Sigma-Aldrich), centrifuged at 13,000 rpm and frozen at −80 °C. All experiments were performed in triplicate.

### 2.2. Selection of Candidate Epigenetic Marks in AD

A set of differentially methylated positions (DMPs) in AD was produced from a methylome dataset generated in a previous study described elsewhere [6]. In brief, the Infinium HumanMethylation450 BeadChip array (Illumina, Inc., San Diego, CA, USA) was performed at the Roswell Park Cancer Institute Genomics Shared Resource (Buffalo, NY, USA) to measure DNA methylation levels in CpG sites (also named *positions*) in a cohort of 26 pure AD cases and 12 controls. A total of 118 AD-related DMPs were identified in the hippocampus of AD cases compared to controls. Here, we selected four of the above-identified DMPs in AD patients compared to controls (absolute β-difference ≥ 0.085 and *p*-value ≤ 0.05) and analyzed them due to their relationship with neurogenesis (Table 1 and Supplementary Figure S1).

### 2.3. DNA Methylation Levels Assessed by Bisulfite Pyrosequencing

Genomic DNA was isolated from frozen cell pellets of basal NPCs and control or Aβ peptide treated NPCs incubated in differentiation media for 9, 19, or 29 days by using the FlexiGene DNA Kit (Qiagen, Redwood City, CA, USA). Next, 500 ng of genomic DNA was bisulfite converted using the EpiTect Bisulfite Kit (Qiagen) according to the manufacturer’s protocol. Primer pairs to amplify and sequence the chosen CpG genomic positions were designed with PyroMark Assay Design version 2.0.1.15 (Qiagen) (Supplementary Table S1) and bisulfite PCR reactions were carried out on a VeritiTM Thermal Cycler (Applied Biosystems, Foster City, CA, USA). Next, 20 μL of the biotinylated PCR product was immobilized using streptavidin-coated Sepharose beads (GE Healthcare Life Sciences, Piscataway, NJ, USA) and 0.4 μM of sequencing primer annealed to purified DNA strands. Pyrosequencing was performed using PyroMark Gold Q96 reagents (Qiagen) on a PyroMark™ Q96 ID System (Qiagen). For each particular CpG, DNA methylation levels were expressed as the percentage of methylated cytosines over the sum of total cytosines. Unmethylated and methylated DNA samples (EpiTect PCR Control DNA Set, Qiagen) were used as controls for the pyrosequencing reaction.

### 2.4. Extension of NXN Gene Methylation Mapping by Bisulfite Cloning Sequencing

Previously bisulfite-converted genomic DNA was used to validate pyrosequencing results. Primer pair sequences were designed by MethPrimer [15] (Supplementary Table S1). PCR products were cloned using the TopoTA Cloning System (Invitrogen, Carlsbad, CA, USA); a minimum of 10–12 independent clones were sequenced for each triplicate, cell condition, and region (Sanger sequencing) [16]. Methylation graphs were obtained with the QUMA software [17].

### 2.5. Neurogenesis Markers mRNA Expression: Analysis by Real-Time Quantitative PCR (RT-qPCR)

Total RNA was extracted from frozen pellets of basal NPCs and the control or Aβ peptide treated NPCs incubated in differentiation media for 9, 19, or 29 days using the RNeasy Mini kit (QIAGEN, Redwood City, CA, USA) following the manufacturer’s instructions. Genomic DNA was digested with DNase I (RNase-Free DNase Set, Qiagen). RNA concentration and purity were determined using a NanoDrop spectrophotometer. Complementary DNA (cDNA) was reversely transcribed from 1000 ng total RNA with SuperScript^®^ III First-Strand Synthesis Reverse Transcriptase (Invitrogen) after priming with oligo-d (T) and random primers. RT-qPCR reactions were performed in duplicate with Power SYBR Green PCR Master Mix (Invitrogen) in a QuantStudio 12 K Flex Real-Time PCR System (Applied Biosystems, Foster City, CA, USA). Sequences of primer pairs were designed using a real-time PCR tool (IDT, Coralville, IA, USA) (listed in Supplementary Table S1). Relative mRNA expression levels of lineage-specific genes in a particular sample were calculated as previously described [18] and the geometric mean of the *ACTB* and *GAPDH* genes used as reference to normalize the expression values.

### 2.6. Immunofluorescence Staining

NPCs were seeded on Nunc™ Lab-Tek™ II chamber slides (Thermo Fisher Scientific, Waltham, MA, USA), coated with CellMatrix Basement Membrane Gel. Cells were either left untreated or treated with Aβ protein fragment 1–42 (50 nM) in differentiation media, as described above. After 9, 19, or 29 days of incubation, cells were fixed with 4% formalin (OPPAC, Noain, Spain) for 15 min; next, they were permeabilized using 0.5% TWEEN^®^ 20 (Sigma-Aldrich) in DPBS and blocked with 10% fetal bovine serum (Sigma-Aldrich) containing 0.5% Tween in DPBS for 30 min at room temperature. Rabbit monoclonal anti-NeuN [EPR12763] (Cat# ab177487, RRID:AB_2532109; 1:300), anti-GFAP [EP672Y] (Cat# ab33922, RRID:AB_732571; 1:300), anti-Synaptophysin [YE269] (Cat# ab32127, RRID:AB_2286949; 1:200) and anti-Ki67 [SP6] (Cat# ab16667, RRID:AB_302459; 1:500) primary antibodies (Abcam, Cambridge, UK) diluted in blocking buffer were added and incubated overnight at 4 °C. After three washing steps, Alexa Fluor^®^ 647 donkey anti-rabbit secondary antibody (Abcam Cat# ab150075, RRID:AB_2752244; 1:500) was added and incubated for 30 min at room temperature in the dark. Following three washing steps, the slides were mounted with ProLong™ Gold Antifade Mountant with DAPI (Molecular Probes, OR, USA). Immunofluorescence images were obtained using a Cytation 5 Cell Imaging Multi-Mode Reader and analyzed with the Gen5™ software (BioTek, Winooski, VT, USA).

### 2.7. Statistical Data Analysis

Statistical analyses were performed with the SPSS version 21.0 (IBM, Inc., Armonk, NY, USA) and GraphPad Prism version 6.00 for Windows (GraphPad Software, La Jolla, CA, USA). We first checked that all continuous variables had a normal distribution using the one-sample Shapiro–Wilk test. Significance level was set at *p*-value < 0.05. Differences between the various time points for mRNA levels of specific genes and percentages of DNA methylation were assessed by one-way analysis of variance (one-way ANOVA) followed by post hoc Tukey’s honestly significant difference test. In cases where the Levene test did not show homogeneity of variance, Welch’s ANOVA followed by Dunnett’s T3 were conducted. Non-parametric data were analyzed using the Kruskal–Wallis test. A paired *t*-test was used to analyze differences in methylation or expression levels of the studied genes between Aβ peptide treated and control groups at each time point. GraphPad Prism version 6.00 for Windows was used to draw the graphs.

## 3. Results

### 3.1. Time-Related Changes in Cultured NPCs during Neural Differentiation

**Figure 1:**
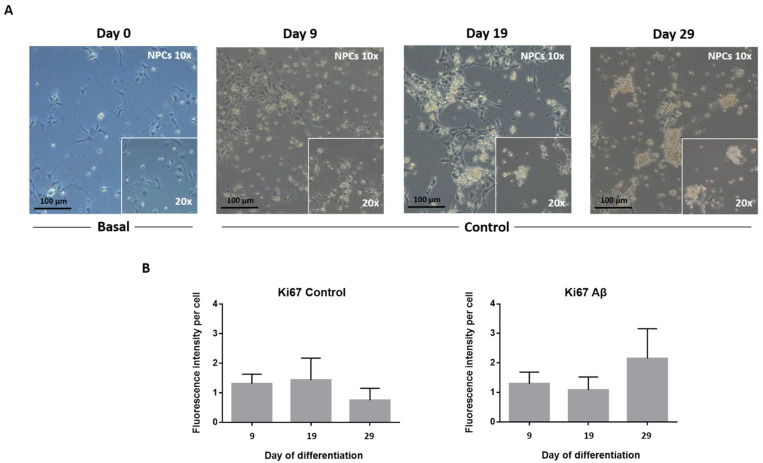
Phenotypic examination of NPCs directed differentiation in culture and Ki67 protein expression. (**A**) Phase-contrast images on days 0, 9, 19, and 29 of basal cells incubated in expansion medium and control cells incubated in differentiation medium (10× magnification with 20× magnification inset lens; the scale bar is 100 µm). (**B**) The graph shows Ki67 proliferation marker expression for control and Aβ-treated NPCs at 9, 19, and 29 days of culture in differentiation medium. Data represent the mean value ± standard error of the mean (SEM).

To determine whether neural differentiation was effectively induced, we first examined any morphological modifications of the cells over time. As shown in Figure 1A, NPCs exposure to differentiation medium led to an increase in the number and length of neuritic extensions, which even connected with the extensions of neighboring cells in comparison with basal cells grown in proliferation medium at Time 0. These changes in cell morphology, typical of cells undergoing differentiation [19,20], were noticed from the first time point (day 9), becoming more evident over time in response to directed neurogenesis.

**Figure 2:**
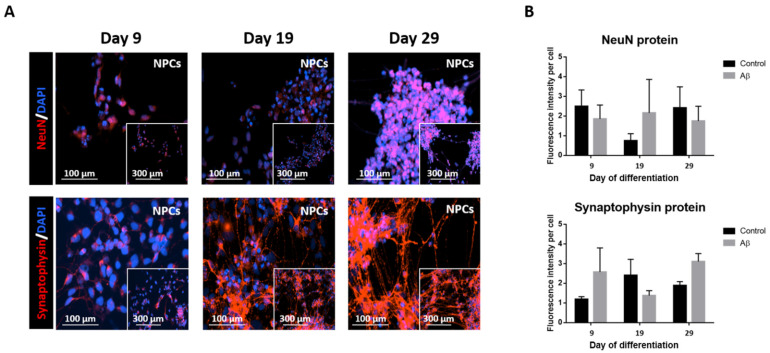
Immunofluorescence staining of NPC differentiation. (**A**) Representative images show NeuN and synaptophysin protein expression on days 9, 19, and 29 in NPCs incubated in differentiation medium (20× magnification (the scale bar is 100 µm) with 10× magnification 4 × 4 montage inset (the scale bar is 300 µm)). (**B**) The graphs show NeuN and synaptophysin markers expression for control and Aβ treated NPCs at 9, 19, and 29 days of culture in differentiation medium. Data represent the mean value ± SEM.

The total cell number in NPC cultures remained steady because of no proliferation, confirmed by unchanged Ki67 protein marker expression in control or exposed to Aβ peptide cells (Figure 1B), which was associated with a gradual boost of cell differentiation. In fact, immunofluorescence (IF) staining revealed neuronal nuclei (NeuN) and synaptophysin protein expression, which mark neurons and synaptic vesicles in the NPC culture (Figure 2).

**Figure 3:**
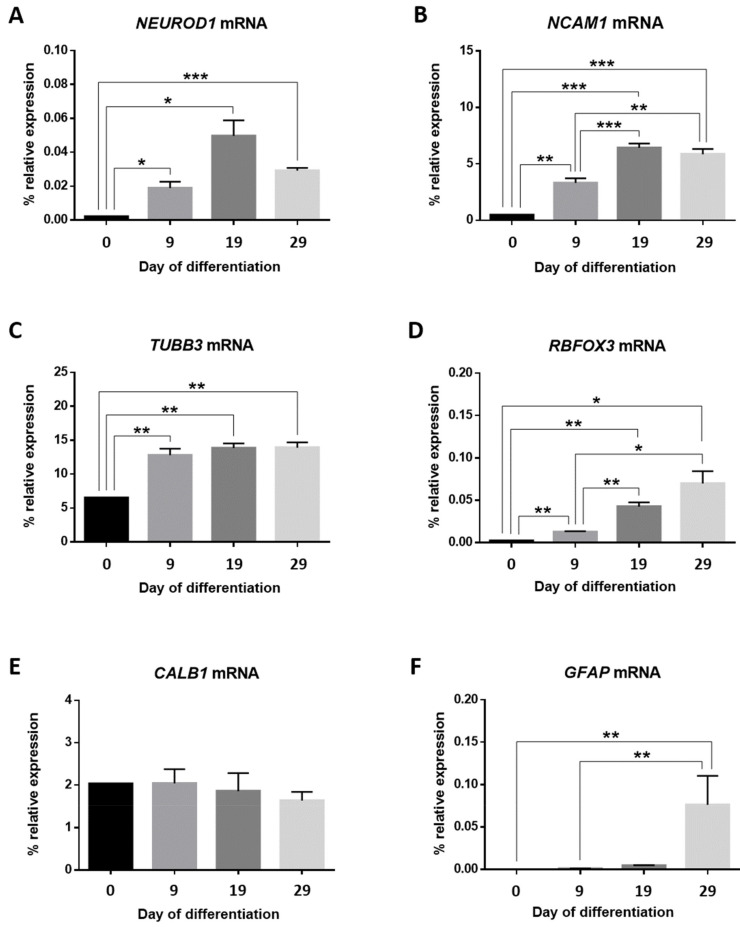
*NEUROD1* (**A**), *NCAM1* (**B**), *TUBB3* (**C**), *RBFOX3* (**D**), *CALB1* (**E**), and *GFAP* (**F**) gene expression profiles. Bar graphs show mRNA percentages of relative expression for each gene relative to the geometric mean of *ACTB* and *GAPDH* housekeeping gene expression for NPCs at each time point of culture. Data represent the mean value ± SEM; * *p*-value < 0.05; ** *p*-value < 0.01; *** *p*-value < 0.001.

To confirm the above observations, we explored if gene expression profiles of different TFs and molecular markers had changed in our *in vitro* model across consecutive stages of driven neuronal differentiation. For that, we measured mRNA expression levels of the Neuronal Differentiation 1 (*NEUROD1*), Neural Cell Adhesion Molecule 1 (*NCAM1*), Tubulin Beta 3 Class III (*TUBB3*), RNA Binding Fox-1 Homolog 3 (*RBFOX3*), Calbindin 1 (*CALB1*), and Glial Fibrillary Acidic Protein (*GFAP*) genes by RT-qPCR (Figure 3). Expression levels of all genes but *CALB1* changed over time.

*NEUROD1* mRNA expression levels of NPCs increased in differentiation medium. Statistically significant increases of mRNA expression for this basic helix-loop-helix (bHLH) TF on days 9 (*p*-value < 0.05), 19 (*p*-value < 0.05) and 29 (*p*-value < 0.001) were observed in comparison to basal cells.

In our *in vitro* model, *NCAM1* mRNA expression overlapped that of *NEUROD1*. We found a statistically significant increase from the addition of differentiation medium to the cell culture (F(3,17) = 31.85, *p*-value = 3.3634 × 10^−7^), which was more pronounced on day 19 (*p*-value < 0.001). Significant differences were also seen between days 9 and 19 (*p*-value < 0.001), days 9 and 29 (*p*-value < 0.01) and between basal cells and any of the other time points: from day 0 to day 9 (*p*-value < 0.01) and from day 0 to day 29 (*p*-value < 0.001).

Once the proliferation medium was changed for differentiation medium, NPCs began to express *TUBB3* mRNA, a gene marker with a key role for proper axon guidance and maintenance. This increase remained constant over time in comparison to basal cells (*p*-value < 0.01). However, no changes were observed between the first, second, and third time points.

*RBFOX3* encodes the NeuN antigen, which has been widely used as a marker for post-mitotic neurons. In our study, *RBFOX3* mRNA expression progressively rises over time, proving the successful achievement of progenitor-to-neuron differentiation. Statistically significant differences in the rise of mRNA expression between day 0 and day 9 (*p*-value < 0.01), day 9 and day 19 (*p*-value < 0.01) and day 9 and day 29 (*p*-value < 0.05) were seen. Likewise, all other differences between any time point with respect to basal cells were also statistically significant: from day 0 to day 19 (*p*-value < 0.01) and from day 0 to day 29 (*p*-value < 0.05).

Regarding *CALB1* mRNA expression, and given that this gene encodes a protein expressed in mature granule cells, no significant changes were detected.

A statistically significant rise in *GFAP* mRNA expression was observed on day 29 in comparison with basal cells (*p*-value < 0.01) and day 9 of differentiation (*p*-value < 0.01). This suggested the presence of NPCs-derived astrocytes in the culture.

None of the neuronal lineage-specific genes showed significant mRNA expression differences between day 19 and day 29.

### 3.2. Assessment of Epigenetic Markers Involved in Neurogenesis in Differentiating NPCs

DNA methylation levels of four neurogenesis-related genes previously found to be altered in the AD hippocampus [6] were quantified by bisulfite pyrosequencing. The same genomic loci identified in the human hippocampus were used to assess DNA methylation levels, corresponding to the genes Contactin-Associated Protein 1 (*CNTNAP1*), *SEPT5-GP1BB* Readthrough (*SEPT5-GP1BB*), T-Box Transcription Factor 5 (*TBX5*), and Nucleoredoxin (*NXN*) (Table 1 and Supplementary Figure S1).

**Figure 4:**
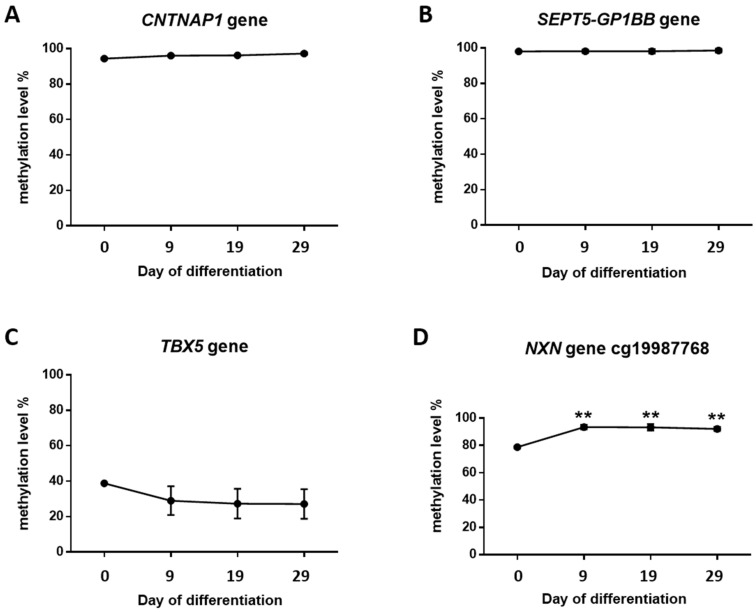
*CNTNAP1* (**A**), *SEPT5-GP1BB* (**B**), *TBX5* (**C**), and *NXN* (**D**) DNA methylation levels in differentiating NPCs. Graphs represent percentages of methylation levels measured by pyrosequencing on days 0, 9, 19, and 29. Vertical lines: SEM. ** *p*-value < 0.01.

No significant differences in DNA methylation levels were observed for *CNTNAP1*, *SEPT5-GP1BB*, and *TBX5* throughout the differentiation process within the time frame of this study (Figure 4A–C).

**Figure 5:**
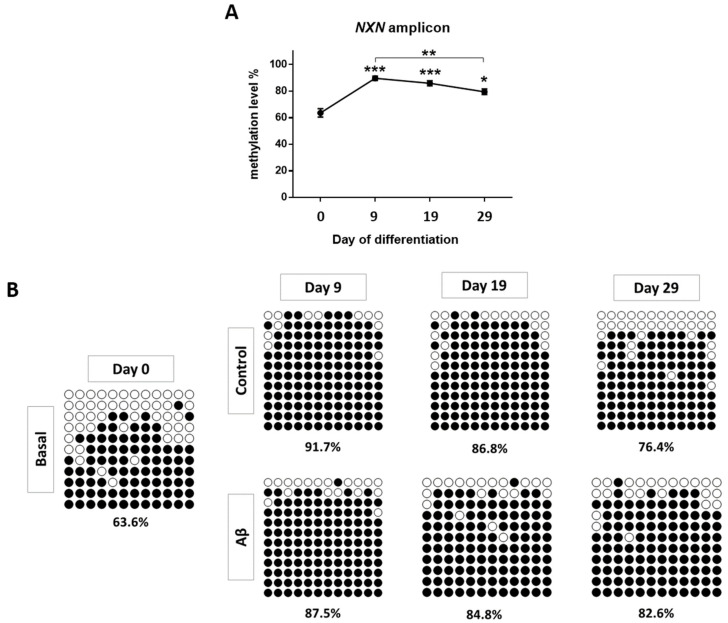
*NXN* DNA methylation levels by bisulfite cloning sequencing. (**A**) Percentages of DNA methylation for *NXN* over time. (**B**) *NXN* extended mapping is illustrated by black/white circle-style figures. Black and white circles denote methylated and unmethylated cytosines, respectively. Each column represents a single CpG site in the examined amplicon, and each line represents an individual DNA clone. Average percentages of methylation for each analyzed sample are indicated at the bottom. * *p*-value < 0.05; ** *p*-value < 0.01; *** *p*-value < 0.001.

Nonetheless, changes in *NXN* methylation levels were observed. Two CpG positions were assessed for the *NXN* gene. For the first one, DNA methylation levels increased on day 9 (*p*-value < 0.01) and were maintained over time; statistically significant differences were also seen on day 19 (*p*-value < 0.01) and day 29 (*p*-value < 0.01) with respect to basal cells (Figure 4D). Regarding the CpG following cg19987768, the pyrogram revealed a similar methylation pattern (day 9 vs. day 0: *p*-value < 0.05; day 19 vs. day 0: *p*-value < 0.01; day 29 vs. day 0: *p*-value < 0.05) (Supplementary Figure S2A). The same differences in methylation levels were observed for both CpGs together (day 9 vs. day 0: *p*-value < 0.001; day 19 vs. day 0: *p*-value < 0.001; day 29 vs. day 0: *p*-value < 0.001) (Supplementary Figure S2B). These findings led us to extend the methylation local mapping for the *NXN* gene using bisulfite cloning sequencing. We confirmed that average DNA methylation levels across all CpG sites for the amplicon were statistically significantly higher at every time point in comparison to day 0 (day 9 vs. day 0: *p*-value < 0.001; day 19 vs. day 0: *p*-value < 0.001; day 29 vs. day 0: *p*-value < 0.05) (Figure 5). Additionally, this approach revealed a decrease in *NXN* DNA methylation levels on day 29, which was statistically significant with respect to day 9 (*p*-value < 0.01).

**Figure 6:**
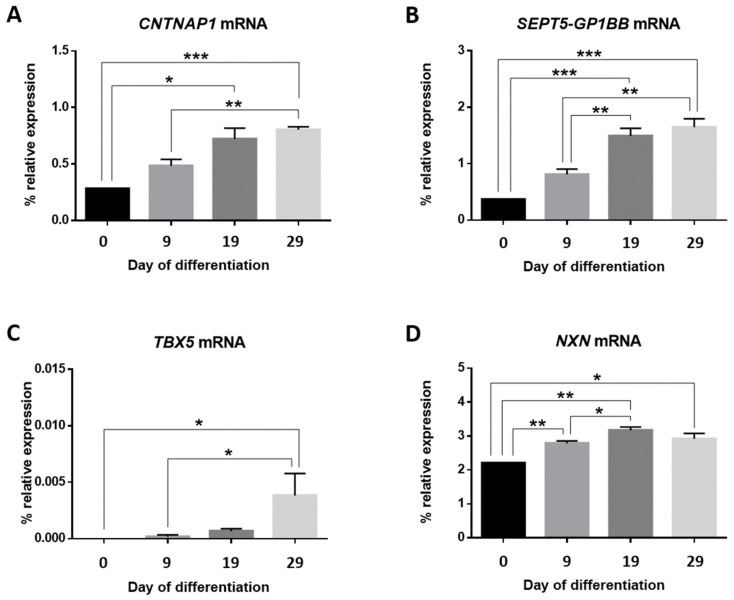
mRNA expression profiles for the *CNTNAP1* (**A**), *SEPT5-GP1BB* (**B**), *TBX5* (**C**), and *NXN* (**D**) genes. Bar graphs represent the percentages of relative mRNA expression for each gene relative to the geometric mean of the *ACTB* and *GAPDH* housekeeping gene expression for NPCs at each time point of culture. Mean values ± SEM. * *p*-value < 0.05; ** *p*-value < 0.01; *** *p*-value < 0.001.

We also measured mRNA expression levels of these markers by RT-qPCR (Figure 6).

*CNTNAP1* mRNA expression levels progressively increased over time with statistically significant differences on day 19 (*p*-value < 0.05) and day 29 (*p*-value < 0.001) in comparison to basal cells. Moreover, significant expression differences were noticed between day 9 and day 29 (*p*-value < 0.01) (Figure 6A).

From day 19, a significant increase in mRNA expression for *SEPT5-GP1BB* was detected (*p*-value < 0.01) and maintained on day 29 (*p*-value < 0.01). Furthermore, mRNA expression on day 19 (*p*-value< 0.001) and day 29 (*p*-value< 0.001) was also significantly higher than for day 0 (Figure 6B).

mRNA levels for the *TBX5* gene increased on day 29 with statistically significant differences in comparison to the cells in culture on day 0 (*p*-value < 0.05) and day 9 (*p*-value < 0.05) (Figure 6C).

Finally, significant differences were observed from the addition of the differentiation medium for the *NXN* gene in terms of gene expression (day 9 vs. day 0: *p*-value < 0.01; day 19 vs. day 0: *p*-value < 0.01); day 29 vs. day 0: *p*-value < 0.05) (Figure 6D). The increase in mRNA expression continued to day 19 (0.384 ± 0.117; *p*-value < 0.05).

Overall, similar transcriptional patterns for the *TBX5* and *GFAP* genes and the *NXN*, *NCAM1* and *RBFOX3* genes during the NPCs culture period, were observed.

### 3.3. Effect of Aβ Peptide Addition on Cultured NPCs during the Stages of Neurogenesis

**Figure 7:**
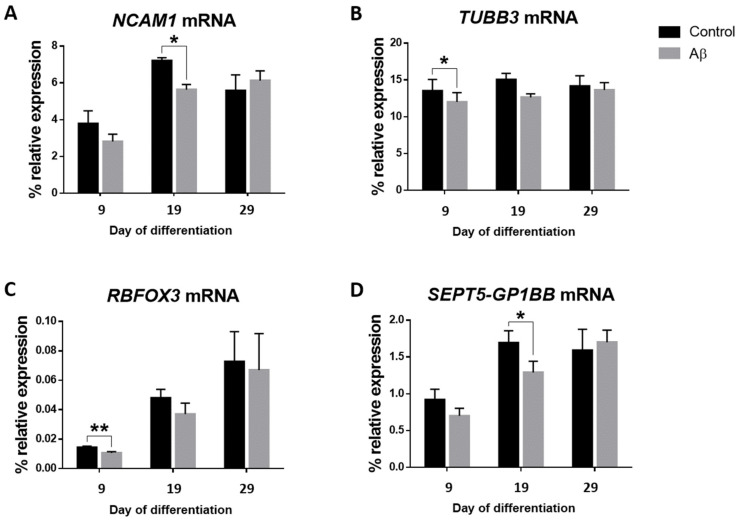
Effect of the addition of Aβ peptide 1–42 during the differentiation period. mRNA expression of the *NCAM1* (**A**), *TUBB3* (**B**), *RBFOX3* (**C**), and *SEPT5-GP1BB* (**D**) genes relative to the geometric mean of *ACTB* and *GAPDH* housekeeping genes expression was determined for the controls and Aβ peptide treated NPCs on days 9, 19, and 29. Vertical lines represent the SEM. * *p*-value < 0.05; ** *p*-value < 0.01.

To mimic the cell environment in AD, we exposed NPCs to Aβ peptide 1–42 once a week during the differentiation period. First, we assessed whether the expression levels of the genes selected to characterize each stage of neurogenesis in culture were altered due to the addition of the Aβ peptide (Figure 7).

We found transient and mild treatment-specific differences in mRNA expression for some of the studied lineage-specific genes. The Aβ peptide reduced *NCAM1* expression (*p*-value < 0.05) on day 19 (Figure 7A), and *TUBB3* (*p*-value < 0.05) and *RBFOX3* (*p*-value < 0.01) expression on day 9 (Figure 7B,C). Interestingly, such decreases occurred at the beginning or in between the studied time window, but these differences were no longer significant at the end time point (day 29).

Next, we assessed how the addition of Aβ peptide affected mRNA expression of neurogenesis-related genes and if the changes had any relationship with their methylation status.

We observed a statistically significant decrease in *SEPT5-GP1BB* mRNA on day 19 (*p*-value < 0.05) (Figure 7D) and an increase in the percentage of DNA methylation with a trend towards statistical significance on day 29 (*p*-value = 0.082) with the addition of the Aβ peptide to the culture.

**Figure 8:**
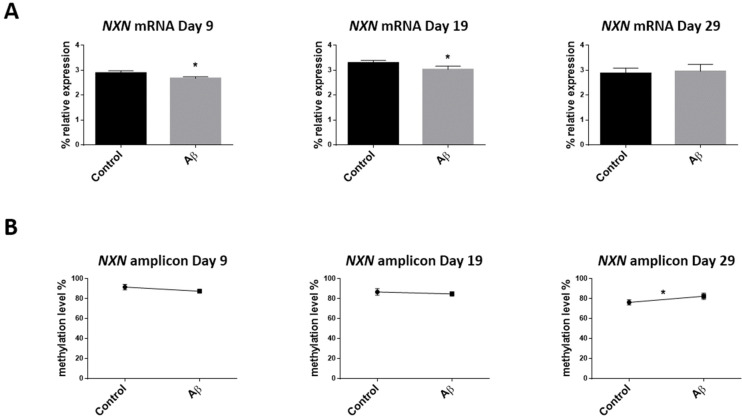
Effect of Aβ peptide 1–42 addition on the *NXN* gene during the differentiation period. mRNA expression relative to the geometric mean of *ACTB* and *GAPDH* housekeeping genes expression (**A**). DNA methylation level in the extended mapping amplicon (**B**) were determined for the controls and Aβ peptide-treated neural progenitor cells on days 9, 19, and 29. Vertical lines represent the SEM. * *p*-value < 0.05.

Finally, the Aβ peptide slightly reduced *NXN* mRNA expression on day 9 (*p*-value < 0.05) which is maintained until day 19 (*p*-value < 0.05) (Figure 8A). *NXN* methylation seems to decrease on day 9 but does not reach statistical significance (*p*-value = 0.11). One possible explanation for this is that the sample size is insufficient to show statistical significance. Interestingly, a rise in the percentage of *NXN* methylation level of Aβ peptide-treated cells was seen on day 29, measuring all amplicon CpG sites (*p*-value < 0.05) (Figure 8B), when the decrease in *NXN* mRNA expression is no longer observed.

## 4. Discussion

**Figure 9:**
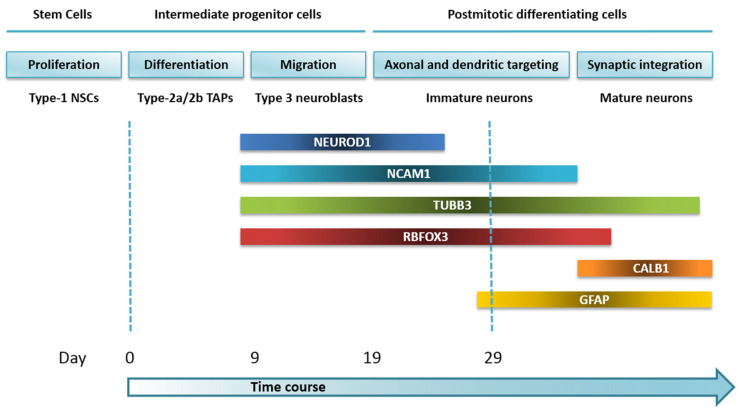
Expression pattern of AHN lineage-specific genes, assessed to characterize our NPCs *in vitro* model. The diagram illustrates *NEUROD1*, *NCAM1*, *TUBB3*, *RBFOX3*, *CALB1*, and *GFAP* gene expression profiles during directed neuronal differentiation for our time window NPCs culture model, based on the developmental stages of AHN within the neurogenic niche of the DG.

To date, a broad overview of the stages of AHN exists. This complex multistep process can be divided into four phases: a precursor cell phase, an early survival phase, a postmitotic maturation phase, and a late survival phase. Type 1 radial glia-like cells (RGLs) represent the NSC population that can differentiate into TAPs (type 2 cells), which initially have a glial (type 2a) and then a neuronal (type 2b) phenotype. Through a migratory neuroblast-like stage (type 3), lineage-committed cells exit the cell cycle ahead of maturation into dentate granule neurons functionally integrated into the hippocampal circuitry [21,22]. Based on cell morphology TFs expression and a set of marker proteins, distinct milestones have been established [21]. In this study, we examined the expression dynamics of key markers in order to characterize a directed human NPCs differentiation model across distinct differentiation stages (Figure 9) to test new AHN epigenetic and expression markers that might be associated with AD.

During stage 1 (proliferation phase), type 1 RGL cells express GFAP. However, no differences in *GFAP* expression are detected until day 19 after the addition of the differentiation medium. This suggests that our *in vitro* NPCs culture window starts after the proliferative phase, during stage 2, when type-2 cells (differentiation phase) lose the GFAP marker [22]. Thus, in contrast to their *in vivo* counterparts in the SGZ of the brain (some authors describe that the *in vitro* expanded NSCs are less neurogenic and mainly biased towards an astrocytic fate upon differentiation [20]), *GFAP* expression on day 19 would correspond to a subset of astrocytes present in our NPCs culture [23].

In stage 3 (migration phase), migrating neuroblasts display the polysialylated form of NCAM (PSA-NCAM), a marker that appears at the late stage of AN and seems to persist in young postmitotic neurons [24]. Accordingly, our results suggest the presence of a plateau between day 19 and day 29 for *NCAM1* mRNA expression. Most PSA-NCAM-positive cells express NeuroD and NeuN, but not GFAP, which supports the abovementioned findings [24]. bHLH TF *NEUROD1* plays an essential role in the differentiation and survival of neuronal precursors in the SGZ. NeuroD1 deletion leads to new granule neurons depletion and their failure to integrate into the DG [25]. In line with findings by Xuan Yu et al. [26], we observed a rise of *NEUROD1* gene expression during our culture time window. Moreover, expression of NeuroD can also be detected in PSA-NCAM-positive cells, precedes it [24], and reaches the highest point in late-stage type 2b and type 3 cells [2]. Once the newly generated neurons become postmitotic, they begin to express the NeuN marker, which is consistent with an earlier *RBFOX3* mRNA expression in our model. We found that *RBFOX3* expression increases until days 19 and 29 of differentiation, showing an expression profile similar to that of *NCAM1*.

Next, cells become postmitotic entering stage 4 (axonal and dendritic targeting). Immature neurons still express PSA-NCAM and, at the same time, can also be marked by NeuN. *TUBB3*, involved in axon guidance and maintenance, is expressed simultaneously; it encodes a class III member of the beta-tubulin protein family, characteristic of early postmitotic and differentiated neurons and some mitotically active neuronal precursors. This is consistent with the increase in *TUBB3* mRNA detected in our model, prior to its translation into protein. *TUBB3* mRNA expression persists in neurons displaying high complexity and electrophysiological properties, such as very low capacitance, high input resistance, depolarized resting membrane potential, and lack of synaptic activity, which show immunoreactivity for NeuN and thus represent postmitotic neurons [24,27].

Finally, mature granule cells establish their synaptic contacts and become functionally integrated into the hippocampus in stage 5 (synaptic integration), expressing calbindin along with NeuN but without co-expressing PSA-NCAM [24]. We do not find variations in *CALB1* mRNA expression within the analyzed culture time window, which may occur later in time. We indeed detect synaptophysin in the IF study on day 29, which suggests that our time window ends early at the synaptic integration phase.

Hence, by culturing NPCs as a monolayer in a medium that accelerates neuronal differentiation by enhancing synaptic activity [14], we achieve a less time-consuming differentiation strategy that resembles the *in vivo* developmental program of human hippocampal DG, which differs from that of the SVZ [28], as we are able to generate developing neurons potentially expressing relevant features of the AHN process.

Once the first objective was accomplished, we evaluated whether a set of AD-related differentially methylated genes targeted specific AHN milestones. These genes had been identified in a previous study of the human hippocampus and annotated as neurogenesis genes following a curated review of the literature [6]. No differences in DNA methylation for the *CNTNAP1*, *SEPT5-GP1BB*, and *TBX5* genes were identified within the period of this study. Only one or two CpGs were analyzed for each gene, those that had been identified as differentially methylated in the hippocampus of AD patients, so changes in DNA methylation may be present in other regions of the gene and may not have been detected with our approach. Still, changes in DNA methylation may occur before or after our time window.

**Figure 10:**
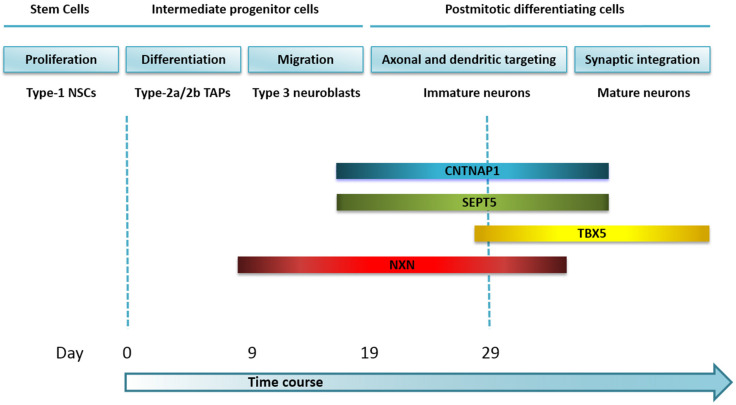
Expression patterns of neurogenesis-related genes evaluated in our *in vitro* model on NPCs. The illustration depicts the expression profiles of the *CNTNAP1*, *SEPT5-GP1BB*, *TBX5*, and *NXN* genes during directed neuronal differentiation of our time window culture model on NPCs, according to the developmental stages of AHN within the neurogenic niche of the DG.

However, it is worth noting that all the above genes undergo mRNA expression changes, suggesting they could be considered potential molecular markers of different AHN stages (Figure 10). Further studies should be carried out to confirm this.

*CNTNAP1* and *SEPT5-GP1BB* mRNA expression levels increase on day 19/29, possibly identifying immature neurons, when axonal and dendritic targeting occurs. Indeed, *CNTNAP1* encodes a type I integral membrane protein that regulates the intracellular processing and transport of contactin to the cell surface [29,30], also known as contactin-associated protein (CASPR), which is present in synapses and interacts with AMPA (α-amino-3-hydroxy-5-methyl-4-isoxazolepropionic acid) glutamate receptors that mediate fast excitatory synaptic transmission in the central nervous system (CNS) [31]. CASPR is an adhesion molecule crucial to forming axoglial paranodal junctions surrounding the nodes of Ranvier in myelinated axons [32].

Known to be a negative regulator of neurite outgrowth in CNS neurons [30], CASPR1 plays an essential role in the timing of neuron and astrocyte development in the mouse cerebral cortex by repressing the transcription of the Notch effector Hes1. In radial glial cells, CASPR1 deficiency delays the generation of cortical neurons and induces the early formation of cortical astrocytes without affecting the number of progenitor cells. Thus, during the neurogenic period, CASPR1 is highly expressed, while during the gliogenic period its expression decreases [32,33]. Moreover, CASPR1 has been reported to be under the regulation of the astrocytic methyl-CpG-binding protein 2 (MeCP2) along with key myelin genes and proteins [34].

For its part, *SEPT5-GP1BB* is originated from naturally occurring read-through transcription between the neighboring *SEPT5* (*SEPTIN5*) and *GP1BB* (Glycoprotein Ib Platelet Subunit Beta) genes on chromosome 22. Inefficient use of an imperfect polyA signal in the upstream *SEPT5* gene causes transcription to continue into the *GP1BB* gene. The Genotype Tissue Expression (GTEx) Project established by the National Institutes of Health (NIH) Common Fund shows the highest median expression of this gene in the brain cortex, but to the best of our knowledge, this is the first study describing *SEPT5-GP1BB* as a possible key marker of temporal specification of cell fate in neurogenesis.

The *TBX5* gene displays the highest level of mRNA expression on day 29. It belongs to a phylogenetically conserved family of genes sharing a common DNA-binding domain, the T-box, which encodes TFs involved in the regulation of developmental processes. Accordingly, it is considered pivotal in the establishment of the cardiac lineage [35]. Moreover, *TBX5* regulates the development of the vertebrate eye [36] and limb skeletogenesis [37]. Here, we observe a statistically significant increase in *TBX5* mRNA at the end time point of our culture (day 29), and therefore, we propose it as a transcriptional candidate marker of postmitotic differentiating cells that may exhibit a peak of expression in the transition of immature to mature neurons.

The most relevant findings of our study relate to the *NXN* gene. At CpG site resolution, *NXN* shows differential methylation at every time point in comparison to basal cells. Moreover, when we extend the mapping and further average across all CpG sites of the amplicon, we confirm these findings and show that peak methylation of *NXN* occurs on day 9. Such curve outlined by the percentage of *NXN* DNA methylation would range from type-2a/2b TAPs to immature neurons, peaking at type 3 neuroblasts. This may allow to discriminate the migration stage of neurogenesis.

Interestingly, the increase in *NXN* methylation is associated with higher mRNA expression levels during our culture time window. DNA methylation at gene promoter regions usually represses gene expression through the recruitment of methylated DNA-binding protein family members, such as methyl-CpG-binding protein 1 (MBD1) and MeCP2. Nevertheless, DNA methylation roles in gene regulation appear complex and multi-faceted and genome structure integration becomes of major importance [38]. In the same way that CG-rich and CG-poor regulatory elements may undergo distinct modes of epigenetic regulation [38], DNA methylation has been linked to gene activation within the transcribed regions and the highest levels of gene body methylation may enhance transcription [39]. Indeed, it is precisely in this region where the studied DMPs are located (Supplementary Figure S1). Thus, DNA methylation has been previously correlated with increased expression in human embryonic stem (ES) cells in an *in vitro*-induced differentiation work [40,41]. Furthermore, gene expression is not only regulated by methylation in the same region, but by other epigenetic mechanisms or methylation in other regulatory areas. Several gene regulatory elements seem to communicate on the same or different chromosomes. Enhancers and insulators participate in this higher-order organization of chromatin [42]. In fact, sequential recruitment of lineage-restricted transcription factors leads to enhancers being activated or maintained in a poised state upon stem cell differentiation [43].

*NXN* is a ubiquitously expressed endogenous antioxidant, member of the thioredoxin antioxidant superfamily [44,45]. In brain sections of mice, there is a predominant neuronal expression of *NXN* in septal nuclei and the hippocampus, in which its deletion results are embryonically lethal, mainly due to cranial defects and deformities [46]. Specifically, immunoreactive signals of *NXN* were found in fibers in the cortex, hippocampus, and cerebellum [46].

In proliferating cells, NXN sequesters dishevelled segment polarity protein 2 (DVL2). Upon the increase in ROS, NXN releases DVL2, relaying the WNT signal to downstream effectors. As a result, cytosolic β-catenin accumulates and shuttles to the nucleus where it drives specific expression of target genes relevant to neuronal differentiation [44,45,47]. NXN also retains a pool of inactive Dvl by preventing the possible interaction of Dvl and kelch-like protein 12 (KLHL12) and its subsequent ubiquitination and degradation, ensuring a prompt activation upon Wnt stimulation [46]. In agreement with this, it has been proved that *NXN* knockdown of SH-SY5Y human neuroblastoma cells increases proliferation and cell cycle reentry [48]. Accordingly, in our *in vitro* model, the increased expression of *NXN* mRNA levels is consistent with the absence of cell proliferation.

The literature points to interactions with further partners that include histone deacetylase 6 (HDAC6), heat shock protein 90 kDa (HSP90), and calcium calmodulin kinase 2a (Camk2a), a postsynaptic kinase crucial for neuronal plasticity [46,48]. Moreover, *NXN* may be implicated in transcriptional regulation, promoting the induction of the TFs CREB (cAMP response element-binding protein), NF*κ*B (nuclear factor kappa B), and AP-1 (activator protein-1) [46].

In the context of AD, it is known that Aβ peptides are generated after the cleavage of APP by γ-secretase in the amyloidogenic pathway [10]. In previous models, the physiological concentration of Aβ peptides in the brain revealed a positive effect on neuroplasticity and learning, showing improved hippocampal long-term potentiation (LTP), while high nanomolar Aβ administration resulted in impaired cognition [49,50], suggesting a hormetic nature [51]. Because low picomolar levels of extracellular concentrations of Aβ in the normal brain have been estimated, in our experiments we chose a concentration of Aβ peptide 1–42 in the nanomolar range (50 nM), added once a week during the 29 days of culture, a single dose determined by the average of concentrations used by Gulisano et al. [52] and Malmsten et al. [53].

It has been reported that the synthetic Aβ peptide 1–42 oligomer decreases human NSC proliferative potential and appears to favor glial differentiation; it reduces neuronal cell fates [10] or suppresses the number of functional human ES cells-derived neurons [54]. Nonetheless, Bernabeu-Zornoza et al. showed that 1 µM monomeric Aβ peptide 1–42 promoted human NSCs proliferation by increasing the pool of glial precursors, without affecting neurogenesis [55]. On the other hand, differentiating neurospheres exposed to fibrillar Aβ decreased neuronal differentiation and induced gliogenesis [54]. The existing controversies may be due to Aβ isoforms, peptide concentrations, aggregation state, administration times, or type of NSCs/NPCs from different species or culture systems used in each experiment [55].

In our work, some of the analyzed genes show a mild decrease in mRNA expression after Aβ 1–42 addition. This transient effect is evident on day 9 or day 19, not occurring on day 29, suggesting that, despite affecting genes involved in the fate of neurogenesis, probably before cells maturation and leading to a decrease in differentiation, the addition of nanomolar concentrations of Aβ is somehow counteracted in the long-term. A time-dependent reversal of the effects of picomolar Aβ on synaptic plasticity and memory had been already seen by Koppensteiner et al., attributable to the enzyme neprilysin, whose levels are reduced with aging and in the brains of AD patients [56]. In fact, a study in which mutant APP was overexpressed to ensure Aβ release exclusively by mature neurons, found neither a positive nor a negative effect in AHN [57]. Hence, our simplistic model may shed light on early AD neurogenesis events, before Aβ deposition cannot be overcome.

A transcriptomic analysis of several human AD profiles demonstrated upregulation of neural progenitor markers expression and downregulation of later neurogenic markers, implying that neurogenesis is reduced in AD due to compromised maturation [58]. Interestingly, the authors showed downregulation of *NCAM1* expression in the hippocampus of early-stage AD, as well as of *NCAM1*, *TUBB*, and *RBFOX3* in late-stage AD, which is in line with our results after the addition of Aβ to the culture. Moreover, Moreno-Jimenez et al. recently provided evidence for substantial maturation impairment underlying AD progression. They identified a decline in doublecortin-expressing cells that co-expressed PSA-NCAM in the DG starting at Braak stage III, followed by a reduction in the expression of NeuN and βIII-tubulin, among others, at some of the subsequent stages of the disease [12].

Our results also show a decrease in *SEPT5-GP1BB* mRNA expression on day 19 when Aβ 1–42 was added to the culture. Again, this suggests that even low levels of Aβ peptide deposit may already have an effect on neuronal fate. For *NXN*, such a decrease in mRNA expression was also observed on day 9 and day 19 cultures. No changes were seen on day 29 when the percentage of methylation levels in the *NXN* amplicon increased in differentiating cells with Aβ 1–42.

Thus, *NXN* emerges as a candidate gene that needs to be further studied to address its ability to determine not only the temporal sequence of neurogenesis but simultaneously the differences in the AD brain due to Aβ peptide deposition.

AHN confers a unique mode of plasticity to the mature mammalian brain. Research in this field requires non-invasive monitoring to understand the lifelong impact [59]. Easier than manipulating NSCs, in part because of the time saving, our NPCs model facilitates studying gene expression levels in an *in vitro* cell culture platform within a human context [60]. Moreover, this straightforward approach may help further understand the alterations affecting specific lineage cell types in presence of the Aβ peptide, including early pathological changes, possibly associated with prodromal phases. On the other hand, other cell types are involved in pathogenesis, particularly microglia, which play a major role, together with neuroinflammation, in the risk of developing AD and its progression. In consequence, co-cultures with other cell types present at neurogenic niches, such as microglia, may be implemented to overcome the limitations presented by the characteristics of an *in vivo* niche environment.

Finally, the development of AHN monitoring methods as biomarkers for cognitive function in live individuals will be crucial to staging AD progress. Moreover, studying the utility of TF reprogramming to preserve endogenous AHN may contribute to cognitive resilience in AD [58]. However, despite the enthusiasm, the prospect of using adult NSCs therapeutically as a regenerative source needs to address neuronal integration and its impact on host mature neural circuits [59]. It will involve strategies to accomplish the NSC pool maintenance, generation of correct neuronal subtypes, suppression of glial fates, and differentiation and survival of immature neurons [2].

## 5. Conclusions

In this work, we present the transcriptional profiles of a number of genes involved in specific stages of the AHN process for a thorough understanding of the lineage-restricted fate during human neuronal differentiation. The addition of Aβ peptide 1–42 to our human NPCs culture model, generates results that are similar to those obtained in human AD samples regarding the expression of the *NCAM1*, *TUBB3*, and *RBFOX3* genes, offering an *in vitro* opportunity to study AHN impairment in the AD context. Considering this approach, the *NXN* gene shows a rise in DNA methylation, the maximum being coincident in time with type 3 neuroblasts and displays differential DNA methylation in immature neurons in presence of the Aβ peptide. Moreover, *CNTNAP1*, *SEPT5-GP1BB*, *TBX5*, as well as *NXN* were revealed as mRNA expression molecular markers for specific stages of AHN. Finally, differentiating NPCs decrease their *SEPT5-GP1BB* or *NXN* mRNA expression at different neurogenesis time points with the addition of the Aβ peptide to the culture.

## Figures and Tables

**Table 1:** Selected differentially methylated positions (DMPs) in AD hippocampus measured by 450 K Illumina BeadChip array. The table shows four DMPs prioritized by beta difference (delta) criteria. Each CpG site was annotated by UCSC hg19 build.

DMPs	Genomic Coordinates		Beta Difference	*p*-Value	Genes
cg16308533	17	40838983	0.118	0.004	*CNTNAP1*
cg04533276	22	19709548	0.117	0.007	*SEPT-GP1BB*
cg18689332	12	114837666	0.106	0.000	*TBX5*
cg19987768	17	750306	−0.162	0.043	*NXN*

## Data Availability

The data presented in this study are available on request from the corresponding author.

## Supplementary Materials

The following supporting information can be downloaded at: https://www.mdpi.com/article/10.3390/cells11071069/s1. Supplementary Table S1. Bisulfite pyrosequencing, bisulfite cloning sequencing and RT-qPCR primers. Supplementary Figure S1. Genomic positions of the CpGs analyzed by pyrosequencing. Supplementary Figure S2. *NXN* DNA methylation levels in differentiating NPCs.

## Abbreviations

Aβ: amyloid β; AD: Alzheimer’s disease; AHN: adult hippocampal neurogenesis; AMPA: α-amino-3-hydroxy-5-methyl-4-isoxazolepropionic acid; AN: adult neurogenesis; ANOVA: two-way analysis of variance; AP-1: activator protein-1; APP: amyloid precursor protein; BDNF: Brain-Derived Neurotrophic Factor; bHLH: basic helix-loop-helix; *CALB1*: calbindin 1; Camk2a: calcium calmodulin kinase 2a; CASPR: contactin-associated protein; cDNA: complementary DNA; *CNTNAP1*: Contactin-Associated Protein 1; *CREB*: cAMP response element-binding protein; DG: dentate gyrus; CNS: central nervous system; DMPs: differentially methylated positions; DVL2: Dishevelled segment polarity protein 2; ES: embryonic stem; GDNF: Glial-Derived Neurotrophic Factor; *GFAP*: Glial Fibrillary Acidic Protein; *GP1BB*: Glycoprotein Ib Platelet Subunit Beta; HDAC6: histone deacetylase 6; HSP90: heat shock protein 90 kDa; IF: immunofluorescence; iPSCs: induced pluripotent stem cells; KLHL12: kelch-like protein 12; LTP: long-term potentiation; MBD1: methyl-CpG-binding protein 1; MeCP2: methyl-CpG-binding protein 2; *NCAM1*: Neural Cell Adhesion Molecule 1; NeuN: neuronal nuclei; *NEUROD1*: Neuronal Differentiation 1; NF*κ*B: nuclear factor kappa-B; NPCs: neural progenitor cells; NSCs: neural stem cells; *NXN*: Nucleoredoxin; PSA-NCAM: polysialylated form of NCAM; PSCs: pluripotent stem cells; *RBFOX3*: RNA Binding Fox-1 Homolog 3; RGLs: radial glia-like cells; RT-qPCR: real-time quantitative PCR; SE: standard error; SEM: standard error of the mean; *SEPT5*: *SEPTIN5*; *SEPT5-GP1BB*: *SEPT5-GP1BB* Readthrough; SGZ: subgranular zone; SVZ: subventricular zone; TAPs: transient amplifying progenitors; *TBX5*: T-Box Transcription Factor 5; TFs: transcription factors; *TUBB3*: Tubulin Beta 3 Class III.
