# Supplementary material for: NXN Gene Epigenetic Changes in an Adult Neurogenesis Model of Alzheimer’s Disease
Source: Cells. 2022 Mar 22;11(7):1069. doi: 10.3390/cells11071069 (PMC8998146; doi:10.3390/cells11071069)
Supplement: Supplementary file 1 [file cells-11-01069-s001.zip › Supplementary Table S1.pdf]

Supplementary Table S1. Bisulfite pyrosequencing, bisulfite cloning sequencing and RT-qPCR primers. The table shows the primer pairs used in the study.

ID	Accesion number	Purpose	Amplicon Size (bp)	Tm	F Primer	Tm2	R Primer
TBX5 pyro	NA	Pyrosequencing	113	60.2	AAGGAGAAGGGAGGAGGTAATAGT	59.3	ATCCCTTTAAAACATCTTCAAAACC
TBX5 seq	NA			47.0	AGGAGGGTAATTGTAGGTAATTT		
TBX5 q	NM_000192	qPCR	135	62.1	GACTTCCTACCAGAACCACAAG	62.1	GGGACCACGGGATATTCTTTAC
NXN pyro	NA	Pyrosequencing	126	57.7	TTGGTAGATGAGGAATGTGAT	60.3	AACCACACTTCCTACTCTTCTAT
NXN seq	NA			45.1	GGTAGGGTGAGTTTTT		
NXN bis	NA	Bisulfite PCR	263	50.7	GTTTTAAATGTTATTATAAATTTTAAGT	58.3	ATTCTACCAAAAAACAAAACTTTCC
NXN q	NM_022463	qPCR	125	61.2	CCAAGTACAAAGCCAAAGAGG	61.4	CAGGATGGTGAGCAAAGGG
CNTNAP pyro	NA	Pyrosequencing	307	61.9	TTTGGTTGGGGATAGAGGGATTT	59.6	CTCCTATACCCAAAAAACCTCATT
CNTNAP seq	NA			42.1	GTTGAAGATTAAGTTTTTTATGTAG		
CNTNAP q	NM_003632	qPCR	143	62.4	CCCCATAGCATCAATATCACCC	62	TCCATCACACGCCCTAAATAC
SEPT5 pyro	NA	Pyrosequencing	134	59.5	AGGGAATGGTGTAAAGTTTAAGT	56.2	CACTACATCCAACAAATAACCAAATA
SEPT5 seq	NA			46.8	GGTGGGTTGATAGGA		
SEPT5 q	NM_002688	qPCR	114	61.9	GATCCATGTATACCAGTTCCCTG	62.4	ACCGTGTTGCTGCCTATAAC
NEUROD1	NM_002500	qPCR	143	61.8	TCCCATGTCTTCCACGTTAAG	61.8	GAGAAGTTGCCATTGATGCTG
NCAM1	NM_001076682	qPCR	128	61.4	CAAGAAAACAGATGAGGGCAC	62.3	TCACAATATTCTGCCTGGCC
TUBB3	NM_006086	qPCR	143	61.8	CGGATCAGCGTCTACTACAAC	62.1	CCAAAGATGAAATTGTCAGGCC
RBFOX3	NM_001082575	qPCR	146	61.8	AGATTTATGGAGGCTACGCAG	62.5	GGTTCCAATGCTGTAGGTCG
CALB1	NM_004929	qPCR	146	62.2	CTGAAGGATCTGTGCGAGAAG	61.3	CTCTAGTTATCCCCAGCACAG
GFAP	NM_002055	qPCR	130	62.4	CATGAAGCCGAAGAGTGGTAC	61.8	AGGTCAAGGACTGCAACTG

Amplified transcripts are identified by RefSeq Accession or GeneBank accesion number. qPCR: quantitative PCR; bp: base pair; Tm: Melting Temperature; NA: not applicable.
