# Supplementary material for: NXN Gene Epigenetic Changes in an Adult Neurogenesis Model of Alzheimer’s Disease
Source: Cells. 2022 Mar 22;11(7):1069. doi: 10.3390/cells11071069 (PMC8998146; doi:10.3390/cells11071069)
Supplement: Supplementary file 1 [file cells-11-01069-s001.zip › Supplementary Figure S1.pdf]

Supplementary Figure S1. Genomic positions of the CpGs analyzed by pyrosequencing. The graph illustrates the track obtained from the Chromatin State Segmentation by HMM from ENCODE/Broad track shown at the UCSC Genome Browser for the genes *CNTNAP1* (A), *SEPT5-GP1BB* (B), *TBX5* (C) and *NXN* (D). CpG islands are represented by isolated green boxes. At the bottom, CpG sites identified in the hippocampus of AD by the Infinium HumanMethylation450 BeadChip array (6) are displayed in bold and underlined. *NXN* following CpG next to cg19987768 included in the pyrogram assay is shown underlined.

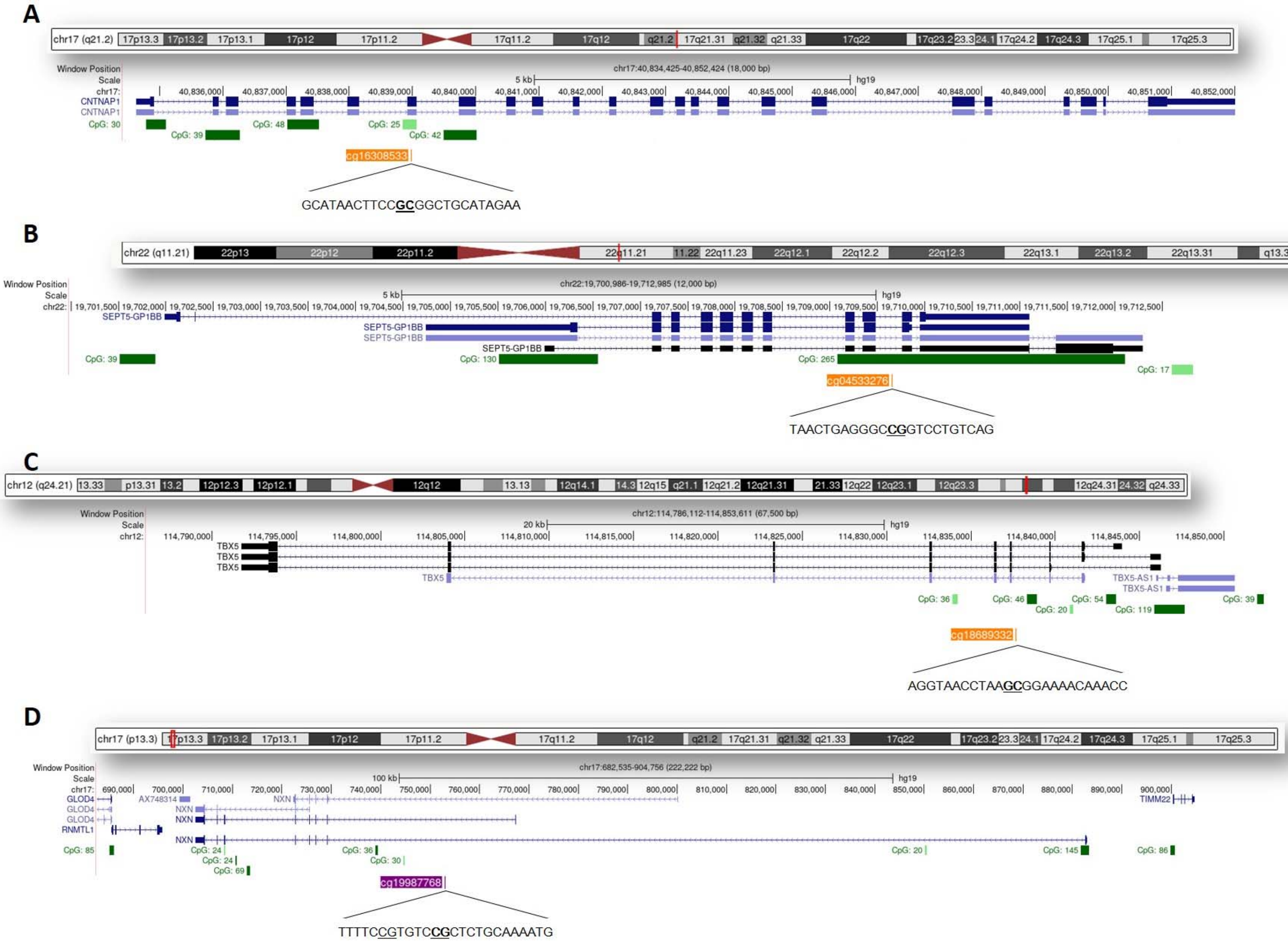

A
chr17 (q21.2)
chr17:40,834,425-40,852,424 (18,000 bp)
hg19
CNTNAP1
CpG: 30
CpG: 39
CpG: 48
CpG: 25
CpG: 42
cg16308533
GCATAACTTCCGCGGCTGCATAGAA
B
chr22 (q11.21)
chr22:19,700,986-19,712,985 (12,000 bp)
hg19
SEPT5-GP1BB
CpG: 39
CpG: 130
CpG: 265
CpG: 17
cg04533276
TAACTGAGGGCCGGTCCTGTCAG
C
chr12 (q24.21)
chr12:114,786,112-114,853,611 (67,500 bp)
hg19
TBX5
TBX5-AS1
CpG: 36
CpG: 46
CpG: 20
CpG: 54
CpG: 119
CpG: 39
cg18689332
AGGTAACCTAAGCGGAAAACAAACC
D
chr17 (p13.3)
chr17:682,535-904,756 (222,222 bp)
hg19
GLOD4
AX748314
NXN
RNMTL1
TIMM22
CpG: 85
CpG: 24
CpG: 24
CpG: 69
CpG: 36
CpG: 30
CpG: 20
CpG: 145
CpG: 86
cg19987768
TTTTCCGTGTCCGCTCTGCAAAATG
