# Supplementary material for: NXN Gene Epigenetic Changes in an Adult Neurogenesis Model of Alzheimer’s Disease
Source: Cells. 2022 Mar 22;11(7):1069. doi: 10.3390/cells11071069 (PMC8998146; doi:10.3390/cells11071069)

Supplementary Figure S2. *NXN* DNA methylation levels in differentiating NPCs. Graphs represent methylation level percentage measured by pyrosequencing at 0, 9, 19 and 29 days for the following CpG next to cg19987768 that the pyrogram assay analyzed (A) and for both CpG1 and CpG2 (B). Vertical lines: standard error of the mean (SEM). *p-value < 0.05; **p-value < 0.01; ***p-value < 0.001.

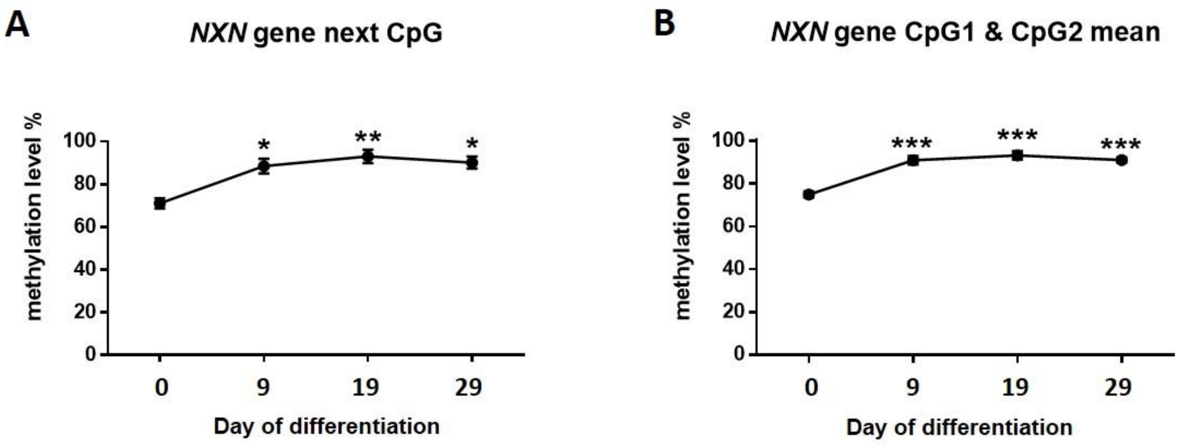

Supplement: Supplementary file 1 [file cells-11-01069-s001.zip › Supplementary Figure S2.pdf]
